# Effectiveness of tDCS at Improving Recognition and Reducing False Memories in Older Adults

**DOI:** 10.3390/ijerph18031317

**Published:** 2021-02-01

**Authors:** Juan C. Meléndez, Encarnación Satorres, Alfonso Pitarque, Iraida Delhom, Elena Real, Joaquin Escudero

**Affiliations:** 1 Department of Developmental Psychology, Faculty of Psychology, University of Valencia, ES 46002 Valencia, Spain; Encarna.Satorres@uv.es (E.S.); erecom@alumni.uv.es (E.R.); 2 Department of Methodology, Faculty of Psychology, University of Valencia, Av. Blasco Ibañez 21, ES 46010 Valencia, Spain; Pitarque@uv.es; 3 Department of Psychology, Faculty of Psychology, Valencian International University, Pintor Sorolla 21, ES 46002 Valencia, Spain; idelhom@universidadviu.com; 4 Hospital General of Valencia, Av. Tres Cruces 2, ES 46014 Valencia, Spain; joaquinescuderotorrella@gmail.com

**Keywords:** transcranial direct current stimulation, true recognition, false recognition, aging, experiment

## Abstract

Background: False memories tend to increase in healthy and pathological aging, and their reduction could be useful in improving cognitive functioning. The objective of this study was to use an active–placebo method to verify whether the application of transcranial direct current stimulation (tDCS) improved true recognition and reduced false memories in healthy older people. Method: Participants were 29 healthy older adults (65–78 years old) that were assigned to either an active or a placebo group; the active group received anodal stimulation at 2 mA for 20 min over F7. An experimental task was used to estimate true and false recognition. The procedure took place in two sessions on two consecutive days. Results: True recognition showed a significant main effect of sessions (*p* < 0.01), indicating an increase from before treatment to after it. False recognition showed a significant main effect of sessions (*p* < 0.01), indicating a decrease from before treatment to after it and a significant session × group interaction (*p* < 0.0001). Conclusions: Overall, our results show that tDCS was an effective tool for increasing true recognition and reducing false recognition in healthy older people, and suggest that stimulation improved recall by increasing the number of items a participant could recall and reducing the number of memory errors.

## 1. Introduction

Human memory is susceptible to distortions, illusions, and false memories that tend to increase during both healthy and pathological aging [1], especially in the face of events that share perceptual or conceptual characteristics. In later life, it is important to minimize these false memories in order to carry out daily activities, such as remembering whether one took their medication, turned off the fire when cooking, closed the door before leaving, or just thought about it. Thus, maintaining a functional episodic memory system is vital for preserving a high quality of life with age, particularly with regard to independent living [2]. Hence, there are obvious benefits if false memories can be reduced temporarily in certain circumstances.

Evidence from injury studies has identified the medial temporal lobe (MTL), particularly the hippocampus and the prefrontal cortex (PFC), as critical brain structures for coding and retrieving episodic memory [3]. Decreased hippocampal volume is associated with reduced memory performance [4], and a decreased anterior, dorsolateral, and ventrolateral PFC is associated with reduced memory capacity [5]. Decreased structure and function of the MTL and PFC are also associated with increased susceptibility to false memories [6]. Older adults with smaller hippocampal volumes generate more false alarms on associative recognition tasks than older adults with larger volumes [7]. In addition, the connective integrity of these two regions is vital for accurate memory coding and retrieval, but there is a reduction in functional connectivity between the MTL and PFC regions with healthy aging [8].

Recently, there has been considerable interest in the use of transcranial direct current stimulation (tDCS) to improve cognition [9,10]. tDCS is a non-invasive technique that elicits constant weak electric currents through the cerebral cortex via electrodes placed on the scalp, flowing from the positively charged anode to the negatively charged cathode. This technique has been shown to modulate excitability in cortical and subcortical tissue and, therefore, may facilitate cell plasticity. The current is thought to modulate the resting membrane potential of neurons depending on the polarity of the electrode such that anodal stimulation induces depolarization of the membrane potential and increases cortical excitability and cathodal stimulation induces hyperpolarization and decreases cortical excitability [11].

A large number of experimental studies [12,13] have demonstrated the efficacy of tDCS in healthy subjects regarding different cognitive tasks, such as associative verbal learning, working memory, selective attention, visual memory, stimulus recall and recognition, and the reduction of false memories [14]. However, a lack of effectiveness of tDCS has been reported, which could be related to the heterogeneity of the parameters of the stimulation [13], such as the area of the stimulation (left lateral cortex, temporal-parietal lobe, etc.), the type of stimulation (anodal, cathodal, or without stimulation (sham)), the amount of current (1 mA, 2 mA, etc.), the type of session (single or repeated) and its duration (15 min, 20 min, 30 min, etc.), the interval between repetitions, the size of the electrode in square centimeters, or the type of design used (between subjects, intra-subject, with or without double-blind control, etc.).

Regarding the effect of tDCS on true recall and recognition, Javadi and Walsh [15] administered anodal or cathodal tDCS over the left dorsolateral prefrontal cortex (dlPFC) during the encoding or recognition of words. With regard to encoding, the data show that only anodal stimulation over the left dlPFC improved memory; in the case of recognition, anodal stimulation was associated with a trend toward improving recognition. These data essentially support the role of the left dlPFC during the encoding and retrieval of words. The effects of tDCS on associative memory have been measured with both recognition and recall tests. The results [16] indicate that significant increases were obtained on recall tests, indicating that tDCS improved the encoding of face–name associations; however, there were no significant effects of stimulation on recognition memory performance. Another study [17] assessed both immediate and delayed stimulation effects of the left dlPFC on associative memory, which was measured in terms of recall and recognition. The authors found no evidence of stimulation-induced recognition memory changes, but improved associative recall was observed. This recall advantage was evident even after a delay of 24 h, suggesting that memory effects persist after a period of consolidation. The authors also point out that these results show that a single session of tDCS while studying (encoding) improved recall performance. In sum, these results seem to indicate that tDCS stimulation applied to the left dlPFC seems to improve true recall, but it has no effect on true recognition.

However, the false memory literature contains few studies and little information. Several authors confirmed the notion that the modulating activity of the anterior temporal lobes (ATLs) with tDCS brain stimulation before or during a given cognitive task is an effective way to change memory processing [14]. They found evidence that anodal tDCS on the left anterior temporal lobes (placed over T3 using the Electroencephalography (EEG) International 10/20 System) is effective at reducing false memories while using a modified version of the Deese–Roediger–McDermott (DRM) paradigm. Anodal left and cathodal right ATLs resulted in a 73% decrease in the formation of false memories. A substantial reduction in false memories has been observed after anode stimulation (over site FT9, according to the International 10-10 System for EEG electrode placement), compared to sham, when using word lists composed of strong associates of the critical words; however, no effect at all emerged when lists were composed of exemplars belonging to the same taxonomic category as the critical lures (categorical lists) [18]. The authors suggest that the left ATL may function as an integration hub when processing associatively related verbal materials in the context of episodic learning.

Given these inconclusive results, the objective of our study was to analyze whether tDCS, through the application of anodal stimulation, was effective at improving true recognition and reducing false memories in healthy older people when using a recognition task to elicit false phonological memories [19]. For the selection of the stimulation parameters, this investigation was based on the most commonly used criteria according to different reviews [13,20].

Traditionally, the study of false memories has been carried out through experimental procedures, where the studied stimuli are semantically related to each other (e.g., tiger, cougar, cat, etc.), which can provoke the false recognition of non-studied critical stimuli that are semantically related to the study list (e.g., panther). However, it is also possible to elicit false memories of critical words (e.g., chair) after studying words that are related to them phonologically rather than semantically (e.g., cheer, hair) [21]. These phonological false memories increase with healthy aging in a similar way to semantic false memories [22,23]. Thus, we proposed an experiment to elicit phonological false memories based on a perceptual manipulation of the stimuli that was implicit for the participants in order to increase the activation of critical words [19]. This adapted procedure [24] mainly consisted of presenting study words formed either from half of the letters in the alphabet (half condition) or from the entire alphabet (entire condition). On the subsequent recognition test, the new words could be formed either from the same letters as the ones studied in the half condition (or critical lures because they were phonologically related to the studied words), distractors formed from the other half of the letters in the alphabet, or distractors formed from the entire alphabet. Therefore, this experimental paradigm, which used a simple study and word recognition task, made it possible to obtain estimates of both true and false recognition (with the latter being operationalized from the false alarms elicited using the critical lures). 

Our idea was to apply this paradigm to healthy older people in two sessions. The materials used in both sessions were different for each subject (and counterbalanced between subjects). Participants were randomly assigned to either a treatment group that received two sessions of electrostimulation through tDCS or a control group that received two sham sessions. This procedure, therefore, allowed us to determine the effectiveness of tDCS applied over site F7 (International 10–20 System for EEG electrode placement) to stimulate the dlPFC by analyzing whether there was an improvement in true recognition or a reduction in false recognition in the treatment group.

## 2. Materials and Methods

### 2.1. Participants

The sample of older adults was composed of 29 people (18 women, 11 men) ranging from 65 to 78 years old (*M* = 68.79, *SD* = 3.33), who belonged to various leisure centers for older adults in the city of Valencia. The Ethical Committee on Human Research of the University of Valencia approved this study. All the participants voluntarily gave their consent to participate, and they reported being in good physical and mental health with no known memory impairments. In this regard, the mean for the older adults on the Mini-Mental State Examination [25] was 29.86 (*SD* = 0.35, range 29–30), revealing no memory impairment. Participants were randomly assigned to receive either tDCS or sham stimulation. The treatment group was composed of 16 older adults (10 women, 6 men) ranging from 65 to 77 years old (*M* = 68.93, *SD* = 3.35); the sham group was composed of 13 older adults (8 women, 5 men) ranging from 65 to 78 years old (*M* = 68.61, *SD* = 3.42). In addition, when comparing the scores for the MMSE [25] between the groups (treatment group = 29.94, sham group = 29.77), no significant differences were observed (*t*(27) = 1.3, *p* > 0.05).

### 2.2. Materials

The half condition included two lists of 50 words each, formed entirely from the following letters of the Spanish alphabet: a, e, u, b, d, g, j, n, r, and z (list A) or i, o, c, f, h, l, m, p, s, t, v, and y (list B). List C (entire condition) contained 50 words formed from the entire alphabet, with the only criterion being that each word had to contain at least one letter from list A and at least one letter from list B. Lists A, B, and C were balanced in terms of mean frequency per two million [26], 93.30 (*SD* = 166.69), 91.28 (*SD* = 129.87), and 92.40 (*SD* = 165.46), respectively, and length, 5.00 (*SD* = 1.20), 4.70 (*SD* = 1.30), and 4.95 letters (*SD* = 1.15), respectively.

### 2.3. Procedure

The experiment took place in two sessions on two consecutive days (one session each day). On day one, participants performed a first study and recognition task with no tDCS stimulation that would serve as a pre-test or baseline (the before condition in Table 1). They were then assigned either to the treatment group or the sham group, receiving either a tDCS or sham stimulation session for 20 min on the second day (24 h later). The stimulation began five minutes before starting the experimental task and continued until the end of the recognition task, which would serve as a post-test (the after condition in Table 1). The experimental task was initiated five minutes after the stimulation started because three minutes of stimulation has been shown to be the minimum time to induce significant after-effect changes in cortical excitability [11].

Each study and recognition task lasted for about 15 min. Each study task consisted of 50 words (presented in a random order for 1.5 s each, with an inter-stimuli period of one second) [24]. Half of the words pertained to the half condition (taken from either list A or list B and counterbalanced across participants; that is, they were formed from half the letters in the alphabet), whereas half the words belonged to the entire condition (from list C; that is, they were formed from all the letters in the alphabet). After the study task, the participants performed the recognition task (self-paced) with 66 words (presented in random order): 15 studied words from the half condition (e.g., from list A), 15 studied words from the entire condition (list C), 12 critical lures formed from the same half of the letters as the studied half condition list (e.g., from list A), 12 distractors from the other half condition list (e.g., from list B), and 12 distractors from the entire condition (list C). The stimuli from the study and recognition tasks were counterbalanced between subjects such that, for example, a participant who studied 25 stimuli from list A and 25 from list C on the first day studied 25 stimuli from list B and the remaining 25 stimuli from list C on the second day. That is, no stimulus was repeated within subjects throughout the two sessions. Because distractors from list A and list B of the entire condition produced similar false alarm (FA) rates, as would be expected based on the equivalence of the two lists, for interpretive simplicity, we decided to average them into only one condition called FA (all the letters in the alphabet; Table 1).

Finally, a debriefing questionnaire asked the participants whether they were aware of any relationships between the words. None of the participants was excluded for this reason, which seems to indicate that our experimental procedure guaranteed an implicit manipulation of the independent variable.

### 2.4. Transcranial Direct Current Stimulation

Starstim tDCS neurostimulator (Neuroelectrics©, Barcelona, Spain) was used to conduct non-invasive tDCS with a constant current intensity of 2 mA. Two 5 × 5 cm rubber electrodes covered with saline-soaked sponges were used to transfer constant direct current, resulting in a current density of 0.08 mA/cm^2^. The anode was placed over site F7 according to the International 10-20 System for EEG electrode placement; this site has been used in previous studies to stimulate the PFC [15,17,27,28]. The cathode was placed over Fp2 in the right supraorbital (rSO) area to minimize its effects on the brain. The stimulation application time was 20 min, with 30 s each for ramping up and ramping down of the current; the same procedure was used for the sham stimulation, but in this case, electric current was only applied in the ramping.

## 3. Results

The overall results of our experiment are shown in Table 1.

In the half condition, the true recognition estimates for each participant were derived by subtracting the proportion of false alarms on words with the same letters, as in the study list (critical words), from the proportion of hits, whereas in the entire condition, the true recognition estimates were derived by subtracting the proportion of false alarms on words with all the letters in the alphabet from the proportion of hits as a way to control the response bias of the participants [29,30].

Regarding these true recognition estimates, a mixed ANOVA with two study conditions (half vs. entire; within subjects) × two sessions (before vs. after treatment; within subjects) × two groups (treatment vs. control; between subjects) showed that the main effect of the sessions variable was significant (*F*(1, 27) = 10.06, *p* < 0.01, *η*^2^*p* = 0.27, indicating that true recognition increased from before the treatment to after it; *M* = 0.52 and *M* = 0.60, respectively), and the session × group interaction was significant (*F*(1, 27) = 33.55, *p* < 0.0001, *η*^2^*p* = 0.55). The remaining main effects and interactions were not significant (*p* > 0.05). Post hoc Bonferroni *t*-tests that were conducted to analyze the significant session × group interaction showed that (a) the true recognition means of the treatment and control groups did not significantly differ before the treatment (*M* = 0.46 and *M* = 0.58, respectively; *t*(27) = 1.85, *p* > 0.05); however, (b) the true recognition mean of the treatment group was significantly higher than the mean of the control group after treatment (*M* = 0.69 and *M* = 0.51, respectively; *t*(27) = 2.76, *p* = 0.01). Furthermore, (c) the control group’s mean before treatment did not significantly differ from its mean after treatment (*M* = 0.58 and *M* = 0.51, respectively; *t*(12) = 1.97, *p* > 0.05); however, (d) the treatment group’s mean after treatment was significantly higher than its mean before treatment (*M* = 0.69 and *M* = 0.46, respectively; *t*(15) = 6.21, *p* < 0.0001).

With regard to the false recognition estimates, we used the relative false recognition index [22,23] by dividing, for each participant, the proportion of false alarms on critical lures by the proportion of hits in the half condition (Table 1) as a way to control the response bias of the participants.

Regarding these false recognition estimates, a mixed ANOVA with two sessions (before vs. after treatment; within subjects) × two groups (treatment vs. control; between subjects) showed that the main effect of the sessions variable was significant (*F*(1, 27) = 9.96, *p* < 0.01, *η*^2^*p* = 0.27, indicating that false recognition decreased from before the treatment to after the treatment; *M* = 0.38 and *M* = 0.29, respectively), and the session × group interaction was also significant (*F*(1, 27) = 16.55, *p* < 0.0001, *η*^2^*p* = 0.38). The main effect of the group variable was not significant (*F* < 1, *p* > 0.05). Post hoc Bonferroni *t*-tests that were conducted to analyze the significant session × group interaction showed that (a) the false recognition means of the treatment and control groups did not significantly differ before the treatment (*M* = 0.43 and *M* = 0.34, respectively; *t*(27) = 1.52, *p* > 0.05); however, (b) the false recognition mean of the treatment group was significantly lower than the mean of the control group after treatment (*M* = 0.21 and *M* = 0.36, respectively; *t*(27) = 2.60, *p* < 0.05). Furthermore, (c) the control group’s mean before treatment did not significantly differ from its mean after treatment (*M* = 0.34 and *M* = 0.36, respectively; *t*(12) = 0.73, *p* > 0.05); however, (d) the treatment group’s mean after treatment was significantly lower than its mean before treatment (*M* = 0.21 and *M* = 0.43, respectively; *t*(15) = 6.21, *p* < 0.0001).

Overall, our results show that tDCS was an effective tool for increasing true recognition and reducing false recognition in healthy older people.

## 4. Discussion

Several studies investigating memory indicate that tDCS can improve true recognition or reduce false recognition. However, few studies have systematically examined the effects of tDCS on both recognition and false recognition in a single experiment. This study aimed to compare the effects of tDCS by comparing an active stimulation group and a placebo group.

Although some studies have demonstrated stimulation-induced memory improvements, as measured by recognition, others have found no improvements. Overall, our results show that tDCS seemed effective at increasing true recognition in healthy older adults in both study conditions, coinciding with other research [15,31], but disagreeing with other research [16,17], which found no improvement in recognition memory after the application of the tDCS.

It has been shown that when applying stimulation with the anode over the left dlPFC, participants performed significantly better on memory accuracy than with cathodal stimulation [31]. The results support the hypothesis that anodal tDCS will lead to higher memory accuracy on the memory recognition task. However, the exact functional role that anodal tDCS plays in improving memory accuracy remains unclear. Memory enhancement derived from stimulating the left dlPFC could have resulted from stronger encoding of target words, better retention of encoded words, or even the engagement of other systems. Moreover, anodal stimulation of the left dlPFC during the encoding phase enhanced memory performance on a later recognition task [31]. Conversely, on a face–name associative memory task, improvements were shown regarding recall but not recognition [16,17]. tDCS applied over F9 during encoding improved associative memory, measured as recall, suggesting that even within the same study, memory effects may be evident only under some testing conditions, specifically those that rely on recollection [16]. The authors speculate that, given the nature of associative memory, tDCS may be effective in promoting cortical connections that support memory in the active stimulation group. After stimulating the dlPFC, it has been suggested that stimulation produces improved memory through both immediate and delayed mechanisms, but that these improvements are only evident under more stringent memory test conditions (recall but not recognition) [17]. One reason is that the dlPFC is thought to play an important role in building relationships between simultaneously presented items at the time of the study, which in turn leads to enhanced associative memory performance.

Neuroimaging and brain damage studies have identified the dlPFC as a key brain region in the ability to recollect specific details, and research indicates that tDCS of the dlPFC during encoding or retrieval can also boost performance [32]. It has also been concluded [33] that, if the dlPFC subserves the cognitively controlled aspects of episodic recollection, then tDCS should also increase the quality of memories, enabling people to more accurately recollect specific details that are associated with studied items and avoid false recollection of erroneous details.

In some studies, false recognition results have shown the positive effects of tDCS in reducing rates of false recall (i.e., producing an item not previously studied) [14]. As in the case of recognition, these results suggest that stimulation improves recall by increasing the number of items a participant can recall and reducing the number of memory errors. Overall, our results clearly show that tDCS also seems effective in reducing false recognition in older people in study conditions similar to previous research [12,14]. Evidence has been found showing that anodal tDCS on the left ATL before the encoding and retrieval phase is effective in reducing false memories, and they confirmed the notion that modulating activity in the ATL, with brain stimulation before or during a given cognitive task, is an effective method for changing memory processing [14]. It has also been found that substantial reductions in false memories were observed after anodal stimulation, compared to sham stimulation, and their results converge by showing that modulating neural activity in the left ATL modifies the pattern of false recognition [12]. Although the results are convergent, some differential aspects must be pointed out in relation to these studies: the tasks that were applied were different and, in our study, the anodal stimulation was on the dlPFC. Previous studies have demonstrated the role of prefrontal regions in forming the inter-item associations that are necessary for successful associative encoding [34]. In our study, participants received stimulation in both the encoding and recall phases, and some studies targeting the dlPFC have reported facilitatory effects when anodal tDCS was administered during online encoding [35,36] or when the stimulation was delivered during retrieval [32].

Positive results may indicate that dlPFC plays an important role in reducing false recognition. Taking into account that in Alzheimer’s disease patients, the temporal zone is the most affected, it is likely that stimulation of the prefrontal zone could produce an improvement in episodic memory, as well as in autobiographical memory, since both are affected [1,37].

One of the limitations of our work could be related to our small sample sizes, which could lead to a lack of statistical power. It would also be worth trying to replicate our results in clinical samples (e.g., patients with mild cognitive impairment, Alzheimer’s disease, etc.). In addition, it should be taken into account that the older adults in the research were younger than 80 years old; therefore, future research could include healthy older adults of more advanced ages and check to see whether the tDCS technique is effective with this population in terms of observing significant results. Future studies should analyze these ideas.

## 5. Conclusions

As a novel finding in the literature, the results showed that tDCS improved the recognition memory of older people, verifying both an increase in true recognition and a decrease in false recognition.

## Figures and Tables

**Table 1:** Means (and *SE*s) of hits (H), false alarms (FA), and estimations of true and false recognition.

		Treatment Group		Control Group	
Conditions	Dependent variables	Before	After	Before	After
Half condition					
	H	0.75 (0.04)	0.83 (0.04)	0.80 (0.04)	0.76 (0.04)
	FA (same letters as in the study list)	0.31 (0.03)	0.17 (0.03)	0.26 (0.04)	0.25 (0.03)
	FA (different letters from the study list)	0.14 (0.02)	0.06 (0.02)	0.15 (0.02)	0.15 (0.03)
	True recognition	0.44 (0.05)	0.67 (0.05)	0.53 (0.05)	0.50 (0.05)
	False recognition	0.43 (0.04)	0.21 (0.04)	0.34 (0.05)	0.36 (0.05)
Entire condition					
	H	0.68 (0.05)	0.80 (0.04)	0.77 (0.05)	0.69 (0.04)
	FA (all the letters in the alphabet)	0.19 (0.03)	0.08 (0.03)	0.14 (0.03)	0.17 (0.03)
	True recognition	0.49 (0.05)	0.71 (0.05)	0.63 (0.06)	0.53 (0.06)

## Data Availability

The data presented in this study are available on request to the authors. The data are not publicly available due to privacy reasons.
